# The axon guidance molecule semaphorin 3F is a negative regulator of tumor progression and proliferation in ileal neuroendocrine tumors

**DOI:** 10.18632/oncotarget.5481

**Published:** 2015-10-02

**Authors:** Julien Bollard, Patrick Massoma, Cécile Vercherat, Martine Blanc, Florian Lepinasse, Nicolas Gadot, Christophe Couderc, Gilles Poncet, Thomas Walter, Marie-Odile Joly, Valérie Hervieu, Jean-Yves Scoazec, Colette Roche

**Affiliations:** ^1^ Centre de Recherche en Cancérologie de Lyon, INSERM U1052, CNRS UMR5286, Equipe «Différenciation endocrine et tumorigenèse», Faculté Laënnec, F-69372 Lyon, France; ^2^ Hospices Civils de Lyon, Hôpital Edouard Herriot, Service Central d'Anatomie et de Cytologie Pathologiques, F-69437 Lyon, France; ^3^ Université Lyon 1, Fédération de Recherche Santé Lyon-Est, ANIPATH, Faculté Laennec, F-69372 Lyon, France; ^4^ Hospices Civils de Lyon, Hôpital Edouard Herriot, Fédération des Spécialités Digestives, F-69437 Lyon, France; ^5^ Université de Lyon, Université Lyon 1, F-69622 Villeurbanne, France

**Keywords:** small intestine neuroendocrine tumor, semaphorin, proliferation, tumor progression

## Abstract

Gastro-intestinal neuroendocrine tumors (GI-NETs) are rare neoplasms, frequently metastatic, raising difficult clinical and therapeutic challenges due to a poor knowledge of their biology. As neuroendocrine cells express both epithelial and neural cell markers, we studied the possible involvement in GI-NETs of axon guidance molecules, which have been shown to decrease tumor cell proliferation and metastatic dissemination in several tumor types. We focused on the role of Semaphorin 3F (SEMA3F) in ileal NETs, one of the most frequent subtypes of GI-NETs.

SEMA3F expression was detected in normal neuroendocrine cells but was lost in most of human primary tumors and all their metastases. SEMA3F loss of expression was associated with promoter gene methylation. After increasing endogenous SEMA3F levels through stable transfection, enteroendocrine cell lines STC-1 and GluTag showed a reduced proliferation rate *in vitro*. In two different xenograft mouse models, SEMA3F-overexpressing cells exhibited a reduced ability to form tumors and a hampered liver dissemination potential *in vivo*. This resulted, at least in part, from the inhibition of mTOR and MAPK signaling pathways.

This study demonstrates an anti-tumoral role of SEMA3F in ileal NETs. We thus suggest that SEMA3F and/or its cellular signaling pathway could represent a target for ileal NET therapy.

## INTRODUCTION

Gastro-intestinal neuroendocrine tumors (GI-NETs) are uncommon neoplasms, but their incidence and prevalence are steadily increasing since the past 30 years [1]. NETs are defined by the proliferation of neoplastic cells retaining most of the characteristics of the normal peptidergic endocrine cells distributed along the digestive tract. Despite their epithelial origin, these cells share several structural and functional properties with neural cells. Like neural cells, they synthesize and secrete neuropeptides. They also express a number of cytoplasmic and surface molecules known to play important roles in the nervous system.

This is the case for several “axon guidance” molecules which have been shown to be constitutively expressed by normal digestive neuroendocrine cells, along with their receptors [2–4]. The putative role of several axon guidance molecules in local invasion and metastatic dissemination has been shown in several tumor types [5, 6]. Moreover, recent evidence suggests that some axon guidance molecules might also be involved in the control of other mechanisms related to tumor growth, such as cell proliferation [7]. However, nothing is known about the involvement of axon guidance molecules in NET progression. In a first attempt to evaluate their possible role, we decided to focus on ileal NETs, because these tumors are one of the most frequent NET subset and that they are usually diagnosed at a late, invasive and even metastatic stage. They are therefore in urgent need of novel biomarkers and therapeutic targets since so far, no medical treatment, including targeted therapies, has shown any significant effect.

The present work is focused on the semaphorin family of axon guidance molecules. Among semaphorins, class 3 molecules are secreted and might therefore be implied in paracrine or autocrine pathways [8]. Secreted class 3 semaphorins are either downregulated or overexpressed in numerous cancers, suggesting the importance of these molecules during tumor progression [9–11]. Among them, semaphorin 3F (SEMA3F) was first described as a tumor suppressor in lung cancer where its expression is lost with the deletion of 3p21.3 region [12–14]. Previous results suggest a possible involvement of the SEMA3F pathway in some NET subsets, such as lung NETs [15]. However, little is known about the functional contribution of SEMA3F to ileal NETs. Here, we show through clinical and experimental *in vitro* and *in vivo* approaches, that SEMA3F expression decreases with tumor progression in human ileal NETs and that this molecule is a negative regulator of neuroendocrine cell proliferation.

## RESULTS

### SEMA3F is expressed in endocrine cells along the small intestine and is lost with tumor progression

**Figure 1:**
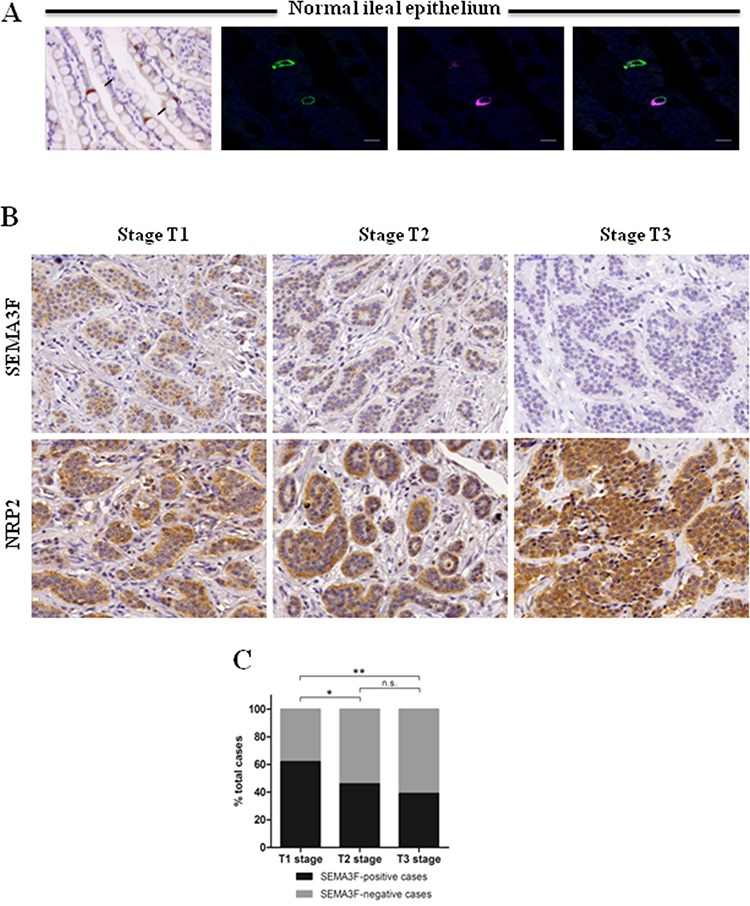
SEMA3F is differentially expressed in human endocrine cells **A.** Immunohistochemical staining of human ileal sections (SEMA3F positivity in brown, black arrows, X200), (*Scale bar*: 25 μm), and confocal analysis of double-immunofluorescence staining (SEMA3F+: magenta; CgA+: green; DAPI) (*Scale bar*: 10 μm). **B.** SEMA3F and NRP2 immunohistochemical staining of human multifocal ileal tumors. **C.** SEMA3F expression according to tumor stage: data represent the percent of SEMA3F positive or negative cases; *P < 0.05, **P < 0 .005.

In human tissue samples, immunohistochemical staining detected SEMA3F expression in the normal intestinal tissue adjacent to the tumor. The expression was restricted to a few cells scattered within the epithelial layer and to ganglion cells in the myenteric plexus. The endocrine nature of normal SEMA3F-positive epithelial cells was confirmed by their co-expression of chromogranin A (CgA), a specific peptidergic endocrine marker (Figure 1A). The SEMA3F-expressing normal cell subsets were then used as internal controls.

Among the 24 primary tumors represented in TMAs, only one showed positive immunoreactivity for SEMA3F. All metastastic deposits represented in the second TMA were negative.

We then studied whole tissue sections of 101 tumors from 38 patients with multifocal NETs, showing different degrees of local invasion. There were 42 tumors limited to the submucosa, staged T1 according to ENETS TNM classification [16], 26 tumors invading the muscularis propria, staged T2, 33 tumors invading beyond the muscularis propria, staged T3. The expression of SEMA 3F clearly decreased with tumor stage, whereas that of its main receptor neuropilin-2 (NRP-2) slightly increased with tumor stage (Figure 1B). The percentage of SEMA3F positive cases was significantly higher in T1 lesions as compared to T3 lesions (*P* < 0.005) (Figure 1C).

**Figure 2:**
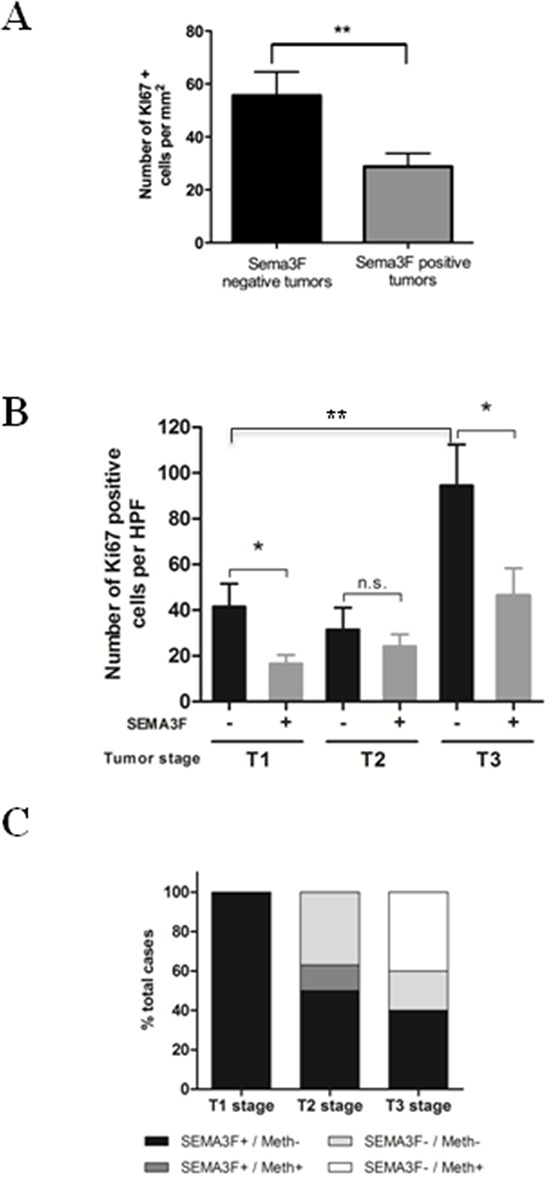
SEMA3F expression level correlates with proliferation rate and promoter methylation **A.** KI67 expression in human multifocal carcinoid tumors: mean ± SEM; **P < 0 .01. **B.** The number of Ki67-positive tumor cells is significantly reduced in SEMA3F-positive tumors compared to SEMA3F-negative tumors within T1 and T3 stages; *P < 0.05 and significantly increases with tumor stage; **P < 0.05. **C.** SEMA3F promoter methylation correlates with loss of SEMA3F expression.

We then explored the possible relation between SEMA3F expression and tumor cell proliferation, as assessed by Ki67 values determined by counting positive cells in areas of highest density of labeled cells [17]. SEMA3F-positive tumors displayed a significantly lower number of Ki67 positive cells per surface unit as compared to SEMA3F-negative tumors (*P* < 0.01) (Figure 2A). Furthermore, SEMA3F-positive T1 and T3 tumors displayed a significantly lower percentage of Ki67 positive cells as compared to T1 and T3 SEMA3F-negative tumors (*P* < 0.05); the number of Ki67 positive cells significantly increased with tumor stage (*P* < 0.01) (Figure 2B). These results suggest that SEMA3F expression is associated with a lower proliferation rate.

### *SEMA3F* gene promoter is methylated in ileal NETs

Previous studies have shown that methylation of a regulatory region in the SEMA3F promoter correlated with loss of SEMA3F expression in some tumoral cell lines [18]. We thus used specific primers designed to amplify this region and assessed methylation status of *SEMA3F* promoter in a subset of 18 ileal NETs from 6 patients with multifocal lesions, including 5 T1, 8 T2 and 5 T3 tumors. An immunohistochemical staining of SEMA3F was performed on control tissue sections of the same tumor. All T1 tumors were unmethylated and positive for SEMA3F. Among the 8 T2 tumors analyzed, 3 were negative for SEMA3F expression but none was methylated. Among T3 tumors, the only 2 tumors positive for SEMA3F were not methylated; 2 out of the 3 SEMA3F-negative cases showed gene promoter methylation (Figure 2B, Supplementary Figure S1A–S1B). Gene promoter methylation could be one of the mechanisms responsible for SEMA3F loss of expression in ileal NETs.

### SEMA3F is differentially expressed in neuroendocrine cell lines

**Figure 3:**
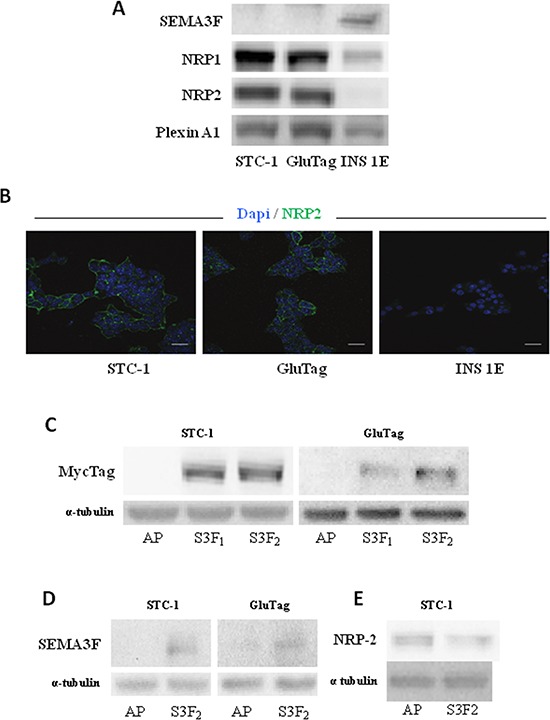
Expression of SEMA3F in intestinal endocrine tumoral cell lines **A.** Immunoblot analysis of SEMA3F, NRP1, NRP2 and PLEXINA1 expression in both STC-1, GluTag and INS-1E cells. **B.** Confocal immunostaining of NRP-2 in STC-1, GluTag and INS-1E cells (NRP-2+: green; DAPI). *Scale bar*: 10 μm. **C.** Immunoblot analysis of MycTag expression in STC-S3F_2_ and GluTag-S3F_2_ clones. **D.** Immunoblot analysis of SEMA3F expression in STC-S3F_2_ and GluTag-S3F_2_ clones. **E.** Immunoblot analysis of NRP2 expression in STC-S3F2 clone.

The expression pattern of SEMA3F and its receptors NRP-1, NRP-2 and PLEXIN-A1 was performed by immunoblotting for three gastroenteropancreatic neuroendocrine cell lines: STC-1 and GluTag, representative of intestinal NETs and known to be highly proliferative and invasive *in vivo*, and INS-1E, representative of pancreatic NETs, poorly proliferative and invasive *in vivo* [19–21]. STC-1 and GluTag cells did not express SEMA3F but exhibited unequivocal expression of both neuropilins and PLEXIN-A1. Conversely, INS-1E cells retained the expression of SEMA3F, NRP-1 and PLEXIN-A1 but did not express NRP-2 (Figure 3A). Confocal laser microscopy analysis confirmed that NRP-2 was expressed at the cell membranes of STC-1 and GluTag cells while the protein was not detectable in INS-1E cells (Figure 3B).

### Generation of stable clones expressing functional SEMA3F

The AP-*SEMA3F*-MYC plasmid was used to generate stable SEMA3F overexpressing STC-1 and GluTag clones (respectively, STC or GluTag -S3F_1_ and -S3F_2_). SEMA3F expression in stable transfected clones was checked by immunoblot using MycTag antibody (Figure 3C) and SEMA3F antibody (Figure 3D). For both cell lines, the SF_2_ clone was chosen for further experiments according to the expression level of Myc. The expression of NRP-2 decreased in STC-S3F2 clone (Figure 3E). Additionally, RBA results suggest that selected clones secreted a SEMA3F protein able to bind the NRP-2 receptor present at the surface of STC-1 and GluTag cells (Supplementary Figure S2).

### SEMA3F overexpression negatively regulates cell viability and proliferation

**Figure 4:**
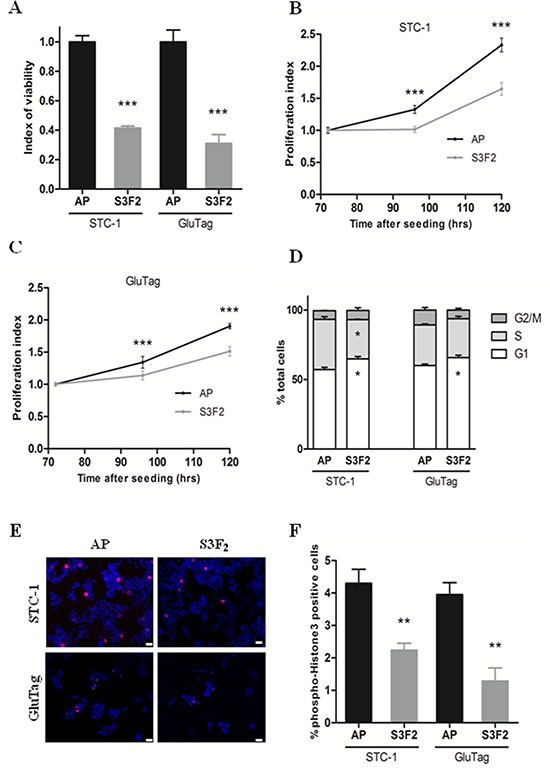
SEMA3F decreases cell viability and proliferation of tumoral cell lines **A.** Cell viability (MTT) assay on STC-S3F_2_ and GluTag-S3F_2_ clones (*n* = 4, ***P < 0 .0005). **B–C.** Kinetics study of cell proliferation using MTT assay at 72, 96, and 120 hours following seeding. (*n* = 3, ***P < 0.0005). **D.** Cell cycle analysis by FACS of STC-1 and GluTag S3F_2_ clones (*n* = 3, *P < 0.05). **E.** Immunofluorescent staining of phospho-Histone H3 (pH-H3 +: red; DAPI) (×200), *Scale bar*: 10 μm. **F.** Quantification of cells positive for pH-H3 staining, (*n* = 3, **P < .005). (mean ± SEM).

In both STC-S3F_2_ and GluTag-S3F_2_ clones, SEMA3F overexpression was associated with a significant decrease of cell viability as measured by MTT assay (*P* < 0.0005) (Figure 4A). STC-S3F_2_ and GluTag-S3F_2_ clones also exhibited a significant decrease in proliferation rate compared to their respective AP clones (*P* < 0.0005) (Figure 4B–4C).

Cell proliferation was further assessed by cell cycle analysis and *p*-HH3 expression. Cell cycle analysis revealed a lengthened G1 phase in STC-1 and GluTag clones overexpressing SEMA3F (*P* < 0.05) (Figure 4D). Furthermore, the number of *p*-HH3 positive cells was strongly reduced in S3F_2_ clones as compared to AP clones (from 4.30% ± 0.43% to 2.24% ± 0.21% for STC, and 3.95% ± 0.37% to 1.29% ± 0.40% for GluTag) (*P* < 0.005) (Figure 4E–4F). No change in the expression of pro-apoptotic cleaved caspase-3 was detected in S3F_2_ clones as compared to controls (Supplementary Figure S3A) indicating that SEMA3F is not acting on caspase-3 dependent apoptosis.

### SEMA3F strongly inhibits tumor cell intrahepatic dissemination *in vivo*

**Figure 5:**
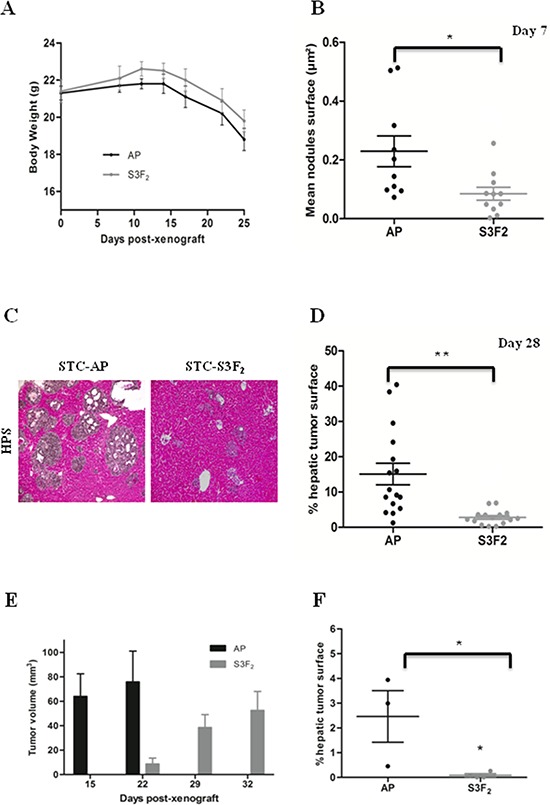
SEMA3F inhibits STC-1 cells intrahepatic dissemination *in vivo* **A.** Body weight curve in STC-AP and STC-S3F_2_ xenografted mice (*n* = 12). **B.** Morphometric analyses of intrahepatic nodules 7 days after intrasplenic injection of STC-AP and STC-S3F_2_ cell lines (*n* = 21, *P < 0.05). **C–D.** Histologic and morphometric analysis at day 28 of intrahepatic nodules following STC-AP and STC-S3F_2_ cell lines grafting. (*n* = 31, **P < 0.005) (×100) *Scale bar*: 100 μm. **E.** Volume comparison of subcutaneous tumors developed after STC-AP or STC-S3F_2_ cells grafting. **F.** Morphometric analyses of intrahepatic nodules 8 weeks after intracaecal graft of either STC-AP or STC-S3F_2_ derived-subcutaneous tumors. (*n* = 7, *P < 0.05); (mean ± SEM).

To explore SEMA3F impact on tumor formation and progression, a mouse model of intrahepatic dissemination after intrasplenic injection of tumor cells was used. We focused on STC-1 clones for *in vivo* studies because the experimental model based on STC-1 has been extensively characterized in our laboratory [22–25]. At sacrifice (day 28 after intrasplenic injection), mice grafted with the STC-S3F_2_ clone exhibited a reduced weight loss compared to animals grafted with the STC-AP clone (Figure 5A).

We then performed morphometric analyses of liver metastatic lesions. At 7 days after tumor cell injection, the overall surface of intrahepatic micronodules was 0.23% ± 0.05% for STC-AP clones as compared to 0.09% ± 0.02% for STC-S3F_2_ clones (*P* < 0.05). This suggests a decrease in liver colonization and/or engraftment (Figure 5B).

At day 28, the percentage of tissue surface occupied by tumor cells was 15.1% ± 3.05% for animals grafted with STC-AP compared to 2.8% ± 0.52% for animals grafted with STC-S3F_2_ clone (*P* < 0.005) (Figure 5C–5D). This significant decrease resulted from a reduction in both the number and the size of intrahepatic nodules (data not shown).

### SEMA3F strongly hampers *in vivo* tumor progression in experimental animal models

A mouse model of intracaecal xenograft was then used because it closely recalls the clinical features and natural history of human GI-NETS, particularly the metastatic dissemination to the liver [26].

First, STC-AP and STC-S3F_2_ clones were subcutaneously injected into nude mice. Subcutaneous STC-AP tumors reached an average volume of 75.89 ± 25.16 mm^3^ at 22 days while the volume of STC-S3F_2_ tumors was only 8.75 ± 4.75 mm^3^ at this stage. STC-S3F_2_ tumors reached a volume of 52.56 ± 15.47 mm^3^ at day 32 (Figure 5E). Fragments from “primary” subcutaneous tumors were grafted on the serous surface of the caecal wall in nude mice. 8 weeks after grafting, intracaecal tumors and liver metastases were obtained in all animals. However, liver metastases developed from STC-S3F_2_ tumors were rare; at morphometrical evaluation, their surface was significantly reduced as compared to controls suggesting that the expression of SEMA3F could affect tumor progression (Figure 5F).

### *In vivo* inhibition of tumor progression by SEMA3F is associated with a decrease in cell proliferation and tumor angiogenesis

**Figure 6:**
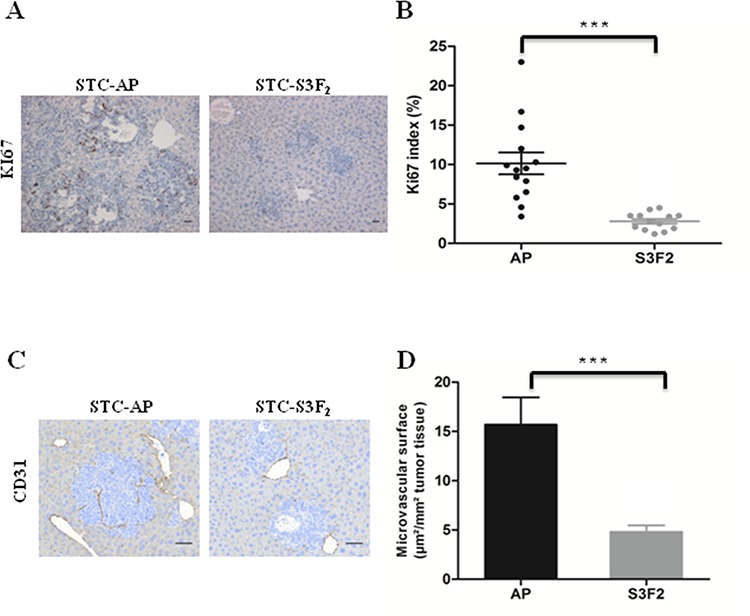
SEMA3F reduces the proliferation rate of tumor cells *in vivo* and inhibits tumor vascularization **A–B.** KI67 immunohistochemical staining and quantification of intrahepatic nodules derived from STC-AP or STC-S3F_2_ intrasplenic grafted cells (*n* = 31, ***P < 0.0005, ×100). *Scale bar*: 50 μm. **C–D.** CD31 immunohistochemical staining and quantification of intrahepatic nodules derived from STC-AP or STC-S3F_2_ intrasplenic grafted cells (*n* = 31, ***P < 0.0005200). *Scale bar*: 25 μm.

To determine whether SEMA3F affect cell proliferation *in vivo*, liver sections from intrasplenic xenografted mice were immunostained for Ki67. Results demonstrated a significant reduction in the percentage of Ki67 positive cells, from 10.14% ± 1.38% in controls to 2.79% ± 0.30% in STC-S3F_2_-induced intrahepatic nodules (*P* < 0.0005) (Figure 6A–6B).

The microvascular network of liver tumor nodules was evaluated after staining with the endothelial cell marker CD31. Results demonstrated that the microvascular surface was dramatically reduced from 15.70 ± 2.76 μm^2^/mm^2^ in controls to 4.80 ± 0.76 μm^2^/mm^2^ tumor tissue in STC-S3F_2_ intrahepatic nodules (Figure 6C–6D).

Expression of cleaved caspase-3 was not modified in STC-S3F_2_ derived intrahepatic nodules as compared to the controls (Supplementary Figure S3B).

### Anti-tumor effect of SEMA3F is associated with alterations of MAPK and mTOR signaling pathways both *in vitro* and *in vivo*

**Figure 7:**
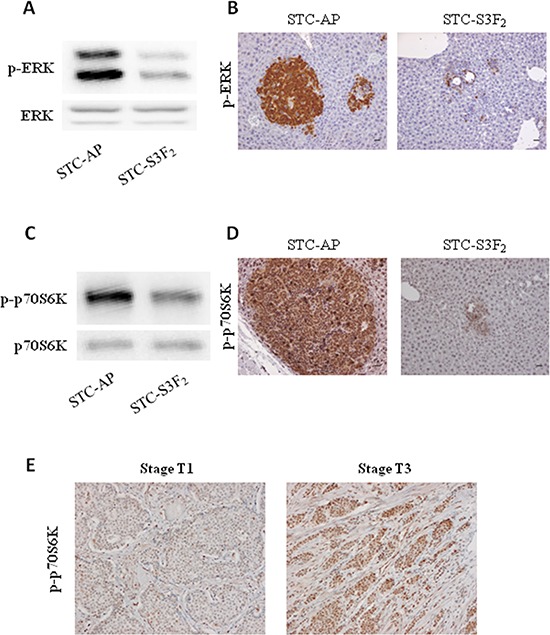
SEMA3F expression decreases activation of MAPK and mTOR signaling pathways **A–C.** STC-AP and STC-S3F_2_ cell lines were subjected to immunoblot analysis of ERK and p70S6K (*n* = 3). **B–D.** Phospho-ERK and phospho-p70S6K immunohistochemical staining on intrahepatic nodules derived from STC-AP or STC-S3F_2_ intrasplenic grafted cells (X400), *Scale bar*: 50 μm. **E.** Phospho-p70S6K immunohistochemical staining on human multifocal ileal tumors (T1 and T3 stages) (X200).

Previous studies have shown that SEMA3F might affect several signaling pathways [27]. To test this hypothesis in our *in vitro* and *in vivo* models, we assessed the expression levels of activated forms of p70S6K and ERK, respectively. The expression of the phosphorylated forms of p70S6K and ERK was reduced in STC-S3F_2_ clone compared to STC-AP clone *in vitro* (Figure 7A–7C). The same observations were made *in vivo* in intrahepatic nodules obtained after intrasplenic injection of these clones (Figure 7B–7D). Furthermore, a strong increase of the expression of phosphorylated form of p70S6K was observed in T3 human lesions compared to T1 lesions (Figure 7E) whereas that of *p*-ERK was not changed (data not shown).

## DISCUSSION

Among class 3 semaphorins, SEMA3F has been studied in several cancer types. The first evidence for a tumor suppressor effect came from lung cancer, in which the region of chromosome 3p21 containing SEMA3F coding gene are commonly deleted [12–14]. Subsequently, SEMA3F expression could be lost early during lung tumorigenesis and its decrease correlated with advanced stages [29, 30]. In the present study, we observed a comparable pattern of expression changes in ileal NETs. SEMA3F was expressed in normal neuroendocrine cells located within the intestinal epithelium, but frequently lost in neoplastic neuroendocrine cells, as shown by our screening of primary invasive tumors and their metastases.

Most patients with ileal NETs present at diagnosis with locally advanced and often metastatic tumors. Therefore, to get further information about the relation between SEMA3F expression and tumor progression, we took advantage of a large series of multifocal ileal NETs. This unique setting allows to correlate SEMA3F expression with local invasion because of the coexistence in the same patient of tumors at different stages of local invasion [31]. We observed a correlation between SEMA3F loss of expression and advanced local invasion. This result was in accordance with previous reports in other tumor types in which SEMA3F loss of expression correlates with advanced disease [29, 32–34]. In addition to the significant correlation observed between SEMA3F expression pattern and local invasion in ileal NETs, we showed an inverse correlation between SEMA3F expression levels and tumor cell proliferative capacities, established by the proliferation marker Ki67 widely used in human pathology. Our results therefore suggest that SEMA3F loss of expression could exert not only a pro-invasive but also a proliferation-inducing effect in ileal NETs.

We then addressed the mechanisms responsible for SEMA3F loss of expression in neuroendocrine tumor cells. We paid attention to the methylation of *SEMA3F* gene promoter, suggested to be a potentially important regulator of *SEMA3F* gene expression [18]. In a significant proportion of the ileal NETs tested in our study, the loss of detectable immunohistochemical expression of SEMA3F protein was associated with gene promoter methylation. This suggests that, as in other tumor types, promoter methylation might be an important mechanism responsible for SEMA3F protein down regulation in tumor cells. However, this is not the only mechanism involved as shown by the presence, in our series, of several cases of SEMA3F negative but unmethylated tumors.

To further explore the functional consequences of SEMA3F loss of expression in neoplastic neuroendocrine cells, we turned to experimental *in vitro* and *in vivo* models. We first aimed to determine the expression profiles of SEMA3F and its preferential receptor NRP-2 in several neuroendocrine tumor cell lines. The highly proliferative STC-1 and GluTag cell lines exhibited very low SEMA3F and high neuropilin-2 expression, while the low proliferative INS-1E cell line, expressed strongly SEMA3F but faintly neuropilin-2. Interestingly, these cellular models retain the apparent correlation between SEMA3F loss of expression and high proliferative capacities.

Our strategy was therefore to evaluate the impact of the re-expression of SEMA3F in STC-1 and GluTag cells *in vitro* and *in vivo*. *In vitro*, both STC-1 and GluTag cells showed a reduced proliferation rate when endogenous SEMA3F expression was restored through stable transfection. This decrease in proliferative activity seemed to be due to a lengthening of G1 phase as if SEMA3F re-expression acted on pathways targeting cell cycle. The next challenge was to assess the *in vivo* effect of endogenous SEMA3F re-expression. In two different *in vivo* experimental models, we observed a significant anti-tumoral effect of the re-expression of SEMA3F in STC-1 neuroendocrine tumor cells. This was shown by a statistically significant decrease in ‘primary’ tumor volumes after grafting in the spleen or in the subcutaneous tissue, as well as in the surface occupied by intra-hepatic liver nodules, obtained either through blood dissemination of intrasplenically grafted tumor cells or after metastatic dissemination from an intracaecal tumor graft. Our results also suggest that the antitumoral effect of SEMA3F re-expression is likely to be multifactorial and might involve a combination of anti-proliferative and anti-angiogenic effects, as shown by the decrease in Ki67 index and in microvascular surface within tumor tissue. In contrast, we could not detect any pro-apoptotic effect in tumor tissue, at least through the evaluation of caspase-3 activity.

We further attempted to uncover the intracellular signaling pathways affected by SEMA3F expression levels. Previous studies in other tumor types have shown that SEMA3F expression is associated with a reduction in activated MAPK signaling [27, 35]. In our models, we were able to demonstrate that mTOR and MAPK signaling pathways were down-regulated in STC-S3F2 cells both *in vitro* and *in vivo*. In human tumors, mTOR pathway appeared strongly activated with tumor stage. A link between these effectors and proliferation has already been demonstrated both by molecular biology experiment but also by using specific inhibitors of mTOR pathway [23]. In our context, SEMA3F could slow proliferation by reducing the activity of mTOR and MAPK pathways, demonstrated to be up-regulated in several cancer types, including neuroendocrine tumors [36]. Taken together, our results suggested that SEMA3F decreased tumor growth through several mechanisms targeting cell proliferation and survival.

In summary, we have showed for the first time that the axon guidance factor SEMA3F was expressed in endocrine cells of the intestinal epithelium and was lost in neuroendocrine tumors arising in the distal small intestine, according to the stage. We also demonstrated that overexpression of SEMA3F reduced the proliferation of neuroendocrine cancer cells *in vitro,* in association with growth inhibition of the xenografted tumors *in vivo*. Therefore, SEMA3F and its signaling pathway could represent a target for neuroendocrine tumor therapy.

## MATERIALS AND METHODS

### Human tissue samples

Tissue samples of GI-NETs were obtained from the collection of digestive neuroendocrine tumors housed in the tumor tissue bank “Tumorothèque des Hospices Civils de Lyon” (Lyon, France), recognized by the French Ministry of Health (DC 2008-72 and AC 2008-73), supported by INCA and member of the BBMRI initiative; the biobank works in strict accordance with French ethical and regulatory issues.

SEMA3F expression was studied in two tissue microarrays and in whole sections of ileal NETs. For expression screening, two tissue microarrays (TMA) were used. The first one contained cores from primary GI-NETs including 24 ileal NETs. The second one contained cores from primary NETs and all their available metastases, including 13 cases of ileal NETs along with their lymph node and liver metastases (34 samples in total); the 13 cases represented in the second TMA were also present in the first one. All 24 tumors were locally advanced and 22 were metastatic to the liver. For further detailed analysis of SEMA3F expression in tumor tissue, samples of 101 tumors from 38 patients with multifocal NETs of the distal small intestine [37], were obtained; we selected these cases in order to directly compare SEMA3F expression between tumors of different local extension originating from the same patient, processed in the same way, and even sometimes present in the same tissue block. 33/38 patients had at least one locally advanced tumor, which determined the overall clinical course; the number of patients with early tumors was therefore not sufficient to make it possible to evaluate the prognostic relevance of SEMA3F expression. Representative whole sections were prepared from each of the 101 tumors.

In all cases, the neuroendocrine nature of the tumor was confirmed by the demonstration of chromogranin A and synaptophysin expression. All studied tumors were well differentiated. Their histological grade was determined according to current recommendations [38]; all tumors were G1 or low G2, with Ki67 index < 5%. For further detailed analyses of proliferation rates in tumor samples, we counted the number of positive tumor cells in an area of 1 mm^2^ selected in the areas of highest density of Ki67 labelled cells.

### Animals

Four-week old female Swiss nu/nu mice (Charles River Laboratories, L'Arbresle, France) were housed and bred in the pathogen free animal facility “AniCan” (Lyon). Experiments were performed in accordance with animal care guidelines of the European Union and French laws and were approved by the local Animal Ethic Evaluation Committee (CECCAPP).

### Intrasplenic xenograft mouse model

The xenografting procedure was as previously described [24]. Briefly, 2.5.10^6^ STC-1 cells were injected into the spleen, from where they disseminated into the liver. The first group (11 mice STC-AP and 11 STC-S3F_2_) was sacrificed at day 7 while the second (15 STC-AP and 16 STC- S3F_2_) was sacrificed at day 28. Both groups were weighted twice a week, and after sacrifice, livers were weighted and prepared for histological analysis.

### Intracaecal xenograft mouse model

The xenografting procedure was performed in 2 steps as previously described [26]. First, subcutaneous tumors were obtained after injection of 2.5.10^6^ STC-AP or STC-S3F_2_ cells into dorsolateral flanks of anesthetized nude mice. Tumor volume was measured at 15, 22, 29 and 32 days after the injection. Then, subcutaneous tumor fragments were orthotopically xenografted into the caecal wall. A total of 7 mice were intracaecally grafted (3 with STC-AP subcutaneous tumors, 4 with STC-S3F2 subcutaneous tumors). Mice were sacrificed 8 weeks after grafting. Intracaecal tumors and livers were recovered.

### Histological analysis and morphometry

Tissue samples were fixed in 10% buffered formalin and embedded in paraffin. For histological examination, 4 μm-thick sections were stained with hematoxylin-phloxin-saffron and observed with a light microscope. The amount of intrahepatic tumor tissue was evaluated by morphometry using Histolab software (Alphelys). The microvascular surface within intrahepatic nodules was assessed by measuring the surface occupied by all structures expressing the endothelial cell marker CD31. The Ki67 index was determined as the percentage of positive cells within intrahepatic nodules.

### Cell culture

The STC-1 and the GluTag cell lines were cultured in DMEM supplemented with 5% (STC-1) or 10% (GluTag) fetal calf serum (FCS), 2 mM glutamine and antibiotics (100 UI/mL penicillin, 100 μg/mL streptomycin) [19, 21]. The INS-1E cell line was in RMPI 1640 supplemented with 10% FCS, 1 mM sodium pyruvate, 50 μM 2-mercaptoethanol, 2 mM glutamine, 10 mM HEPES and antibiotics [20].

### Antibodies

For immunoblot, antibodies to SEMA3F (ab39956) and NRP-1 (ab81321) were purchased from Abcam (Cambridge, UK). Antibodies to NRP-2 (#3366), PLEXIN-A1 (#3813), Myc-Tag (#2276), phospho-ERK (#4370), ERK (#9102), phospho-p70S6K (#9234), p70S6K (#9202) and cleaved caspase-3 (#9664) were from Cell Signaling Technology (Beverly, MA, USA) and the antibody to α-Tubulin (T6074) was from Sigma-Aldrich (St. Louis, Mo, USA).

For immunohistochemistry, the antibody for SEMA3F (HP035008) was purchased from Atlas Antibodies. Anti-phospho-p70S6K (sc-7984) was from Santa Cruz Biotechnology (Santa Cruz, CA, USA). Anti-chromogranin A (M0869), anti-human Ki67 (MIB1 clone, M7240) and anti-mouse Ki67 (TEC-3 clone, M7249) were from Dako (Glostrup, Denmark). Anti-CD31 antibody was from AnaSpec (#53332) (Fremont, CA, USA). For immunofluorescence, the antibody to phospho-Histone H3 (pH-H3) was from Cell Signaling Technology (#9706).

### Methylation specific PCR (MSP)

DNA from tumor tissues was extracted from 18 formalin-fixed paraffin-embedded ileal NETs according to manufacturer's instructions (Master Pure DNA and RNA purification kit, Epicentre Biotechnologies, Madison, USA). DNA modification was performed according to manufacturer's instructions (Epigentek, Farmingdale, USA) then MSP was performed as described by Herman *et al*. [39].

### Immunohistochemistry

Both human and mouse tissue sections were subjected to antigen retrieval, then treated with 3% H_2_O_2_ for 10 minutes. An enhanced streptavidin-biotin staining procedure was followed: sections were incubated with the appropriate biotinylated antibody for 30 minutes at room temperature; revelation was carried out using the EnVision™ detection kit (Dako) according to manufacturer's instructions; sections were then counterstained with Meyer's hematoxylin.

### Immunofluorescence staining

Tissue sections and cell lines grown on glass coverslips were incubated with the primary antibodies respectively for 30 minutes at room temperature or overnight at 4°C. Both cells and sections were then incubated with secondary Alexa Fluor 488 or −568-conjugated antibodies (Life Technologies, Carlsbab, CA, USA) for 1 hour and counterstained with DAPI. Finally, pictures were taken using a Leica SP5X confocal laser scanning microscope or a Zeiss inverted microscope Axio Observer D1.

### Protein analysis

Cells were seeded and maintained 72 hours under normal culture conditions, then lysed in RIPA buffer plus protease and phosphatase inhibitors (Santa Cruz Biotechnologies). The membranes were hybridized with primary antibodies overnight at 4°C. Immunodetection was performed using electrochemiluminescence (ECL; Covalab, Villeurbanne, France) and the ChemiDoc XR5 machine (Bio-Rad, Marnes la Coquette, France).

### Generation of stable SEMA3F-overexpressing cells

The SEMA3F expression plasmid AP-*SEMA3F*-MYC and the corresponding empty vector pSectag-AP were kindly provided by Dr. David Ginty (Howard Hughes Medical Institute, USA) and Dr Joëlle Roche (University of Poitiers, France). Briefly, the full-length SEMA3F cDNA fragment lacking stop codons was ligated into the pSectag expression vector (Life Technologies). The full length SEMA3F was placed between an APtag sequence at N-terminal and a MYC tag sequence at the C-terminal ending [40]. Both AP-*SEMA3F*-MYC and pSectag-AP plasmids were transfected into STC-1 and GluTag cells by using the SuperFect Transfection Reagent and selected for stable expression with 300 μg/mL zeocin (Life Technologies).

### Receptor binding assay

We used receptor-binding assay (RBA) staining method to demonstrate SEMA3F ectodomain binding within our cell lines. Selected stable clones containing the pSectag-AP empty vector or the AP-*SEMA3F*-MYC vector were grown in 25 cm^3^ tissue flasks with medium devoided of FCS and antibiotics. Then, the culture supernatant was recovered and concentrated by size exclusion filtration by using a 30 kDa Amicon Ultra centrifugal filter unit (Millipore). RBA was performed following the procedure described by Brennan *et al.* [41].

### Survival, proliferation and cell cycle analysis

Cell survival and proliferation were assessed by MTT method. The proliferative capacities of transfected cells were evaluated by phospho-Histone H3 (pH-H3) immunostaining. Cell cycle analysis was performed using propidium iodide-based Cycletest™ Plus-DNA Reagent Kit (BD Biosciences, Le Pont de Claix, France) and Canto II cytometer.

### Statistical analysis

All data were mean values of at least three individual experiments and were expressed as mean ± SEM. Differences between means were compared by unpaired two-tailed student's *t*-test or Chi-squared test. A *P* value inferior to 0.05 was considered as significant.

## SUPPLEMENTARY FIGURES


