# Supplementary material for: The axon guidance molecule semaphorin 3F is a negative regulator of tumor progression and proliferation in ileal neuroendocrine tumors
Source: Oncotarget. 2015 Oct 2;6(34):36731–45. doi: 10.18632/oncotarget.5481 (PMC4742207; doi:10.18632/oncotarget.5481)
Supplement: Supplementary file 1 [file oncotarget-06-36731-s001.pdf]

SUPPLEMENTARY FIGURES

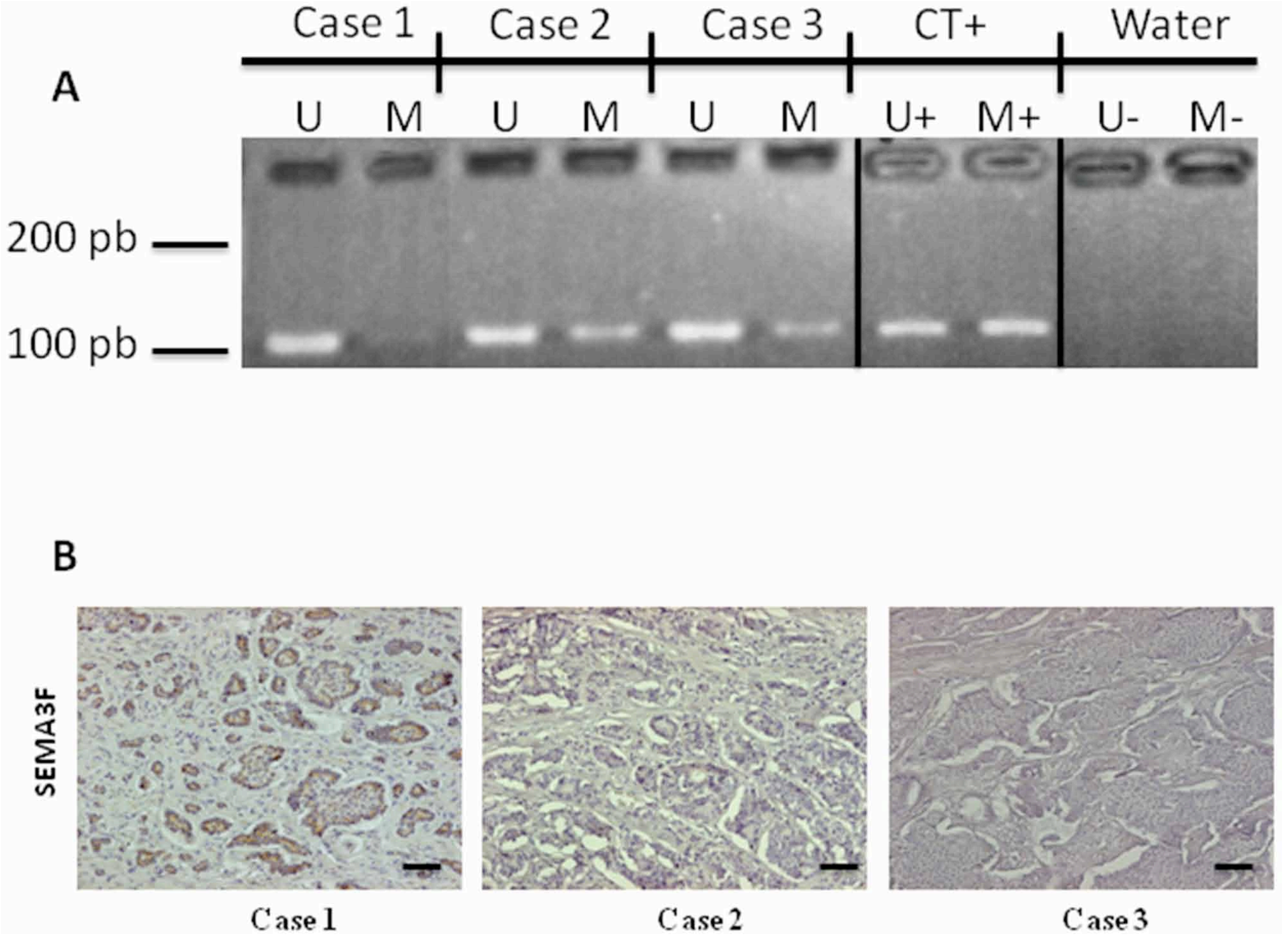

Supplementary Figure S1: SEMA3F loss-of-expression is associated with promoter methylation in human multifocal NETs. A. MSP assay on 3 human tumors (cases 1–3). U = unmethylated, M = methylated. U+ and M+ are positive controls (CT+) for Unmethylated or Methylated DNA. Case 1 is a T1-staged multifocal NETs while Cases 2 and 3 are T3 tumors. **B.** SEMA3F immunohistochemical staining of cases 1, 2 and 3 (X100). *Scale bar*: 50 µm.

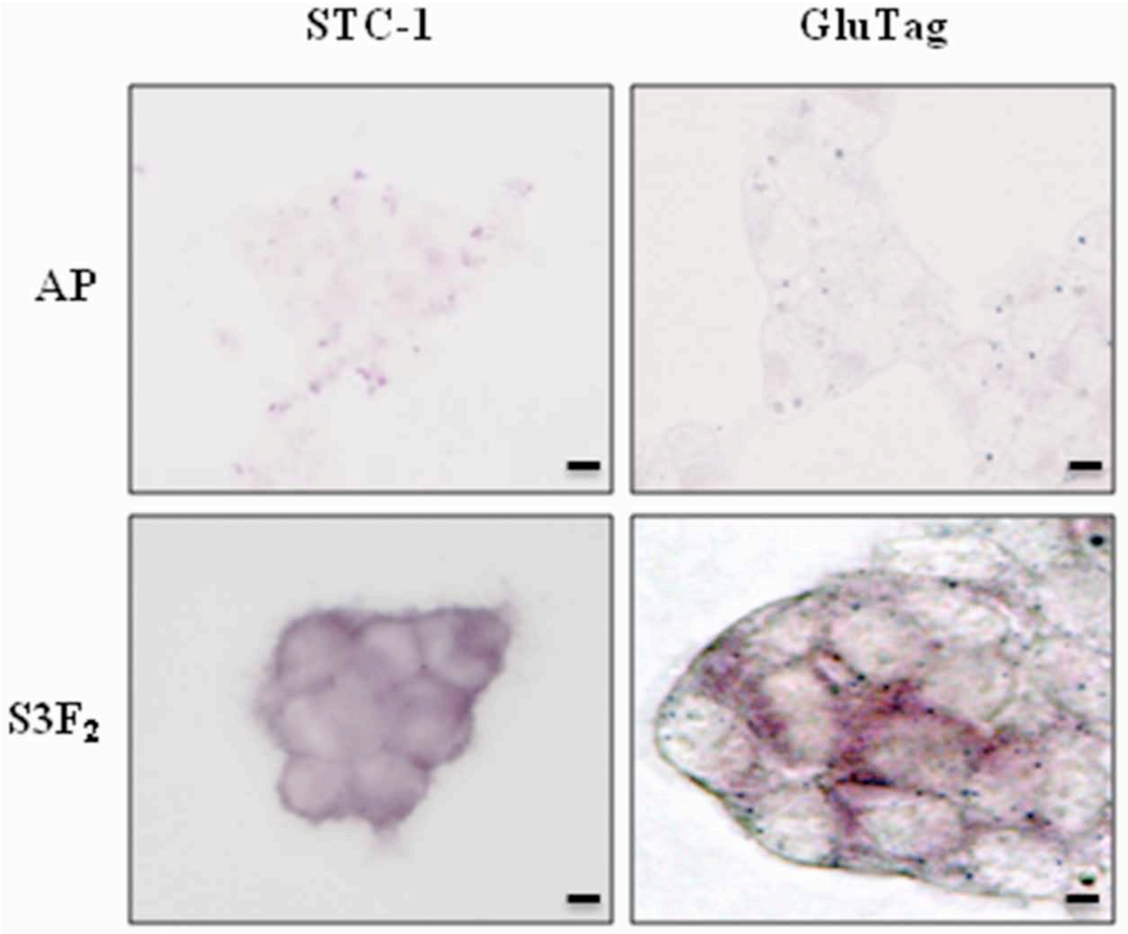

Supplementary Figure S2: Receptor-binding assay on STC-1 and GluTag wild-type cells (positive staining in purple-brown), (X400). *Scale bar*: 10 μm.

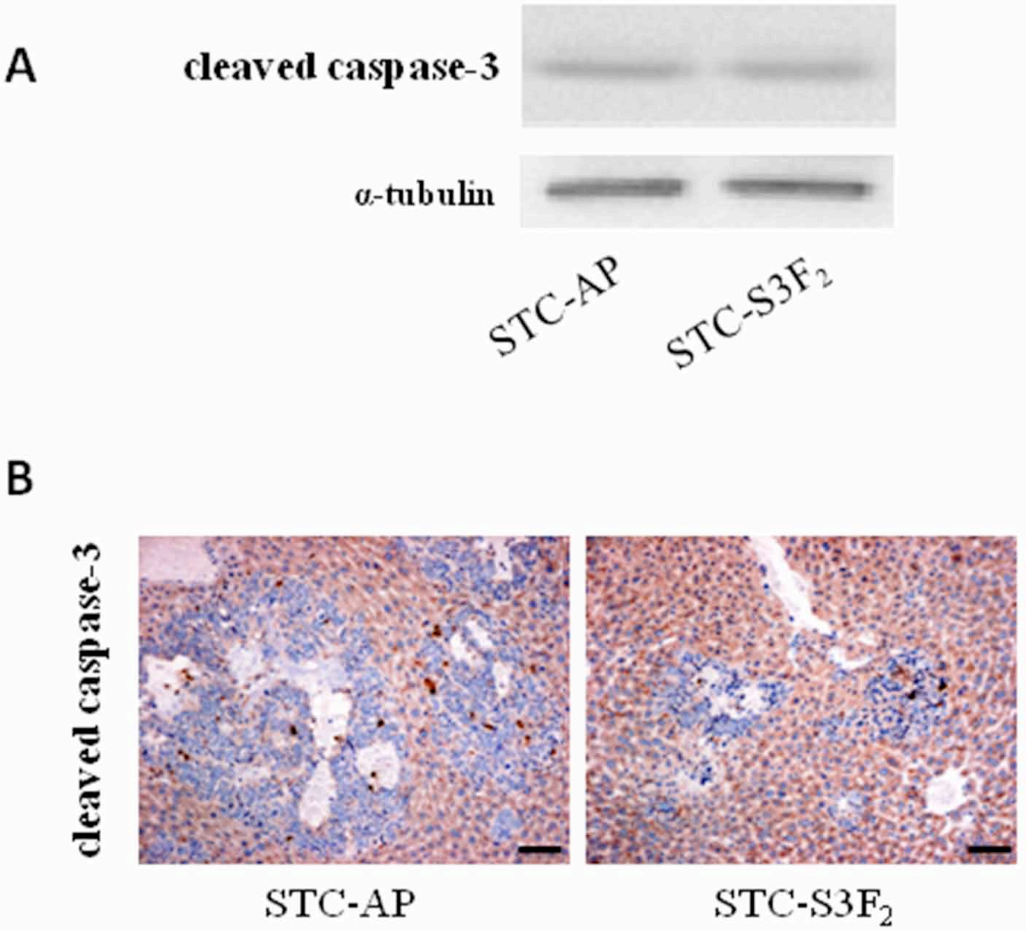

Supplementary Figure S3: **A.** Activated Caspase 3 immunoblotting of STC-AP and STC-S3F2 cells (n = 3). **B.** Activated Caspase 3 immunohistochemical staining of intrahepatic nodules derived from STC-AP or STC-S3F2 intrasplenic grafted cells (×200). *Scale bar*: 25 µm.
